# Prognostic efficacy of lymph node parameters in resected ampullary adenocarcinoma based on long-term follow-up data after adjuvant treatment

**DOI:** 10.1186/s12957-024-03587-z

**Published:** 2024-11-21

**Authors:** Namyoung Park, In Rae Cho, Sang Hyub Lee, Joo Seong Kim, Jin Ho Choi, Min Woo Lee, Woo Hyun Paik, Kwang Ro Joo, Ji Kon Ryu, Yong-Tae Kim

**Affiliations:** 1 Department of Gastroenterology, Kyung Hee University College of Medicine, Kyung Hee University Hospital at Gangdong, Seoul, Republic of Korea; 2 Department of Internal Medicine and Liver Research Institute, Seoul National University College of Medicine, Seoul National University Hospital, 101 Daehak-Ro, Jongno-Gu, Seoul, 03080 Republic of Korea; 3 Department of Internal Medicine, Dongguk University College of Medicine, Dongguk University Ilsan Hospital, Goyang-Si, Republic of Korea

**Keywords:** Ampullary adenocarcinoma, Lymph node metastasis, Prognosis

## Abstract

**Background:**

Lymph node (LN) metastasis is an important prognostic factor in the ampulla of Vater (AoV) adenocarcinoma. Various LN parameters have been proposed, but their prognostic efficacy has not been compared in the same population. We aimed to evaluate the prognostic values of LN parameters in AoV adenocarcinoma patients who underwent surgical resection and adjuvant treatment based on the long-term follow-up data.

**Methods:**

A total of 86 patients with surgically resected AoV adenocarcinoma followed by adjuvant treatment were analyzed. We evaluated the prognostic values of various LN parameters such as pathologic N stage, number of metastatic regional LN (LNN), LN ratio (LNR), and log odds of positive LNs (LODDS). Each LN parameter was separately analyzed using Cox regression models with the same confounders.

**Results:**

The median follow-up period was 69.4 months, and the median overall survival (OS) was 114 months. The median number of dissected LNs is 15, with an interquartile range of 8 to 25. In the univariable analyses, all LN parameters showed significant prognostic efficacy for OS, disease-free survival (DFS), and distant metastasis-free survival (DMFS). In the multivariable Cox regression analyses, LNN ≥ 2 was a statistically significant prognostic factor for OS (hazard ratio (HR) 2.10, 95% confidence interval (CI), 1.11–3.97; *p* = 0.022), DFS (HR 2.51, 95% CI 1.28–4.93; *p* = 0.007), and DMFS (HR 2.74, 95% CI 1.39–5.41; *p* = 0.004). LNR showed significant prognostic performance for DFS (HR 2.35, 95% CI 1.23–4.50; *p* = 0.010), and DMFS (HR 2.26, 95% CI 1.17–4.35; *p* = 0.015). N stage showed significant prognostic performance in DFS (HR 1.55 for pN1; *p* = 0.243 and HR 4.31 for pN2; *p* = 0.003), DMFS (HR 1.46 for pN1; *p* = 0.323 and 4.59 for pN2; *p* = 0.002). LODDS and the presence of LN metastasis, did not demonstrate significant prognostic value across survival outcomes.

**Conclusions:**

LN parameters showed good long-term predictive performance in AoV adenocarcinoma patients treated with curative resection and adjuvant treatments. Among LN parameters, LNN ≥ 2 showed better prognostic value than others. Further large-scale studies are needed to validate the clinical usefulness of various LN parameters.

**Supplementary Information:**

The online version contains supplementary material available at 10.1186/s12957-024-03587-z.

## Introduction

Ampulla of Vater (AoV) adenocarcinoma is a rare gastrointestinal malignancy. The incidence rate is 0.49 per 100,000 population, accounting for only 0.2–0.5% of all gastrointestinal malignancies [1]. Compared to other pancreatobiliary malignancies, AoV adenocarcinoma is known to have a better prognosis because symptoms such as jaundice appear at a relatively earlier phase of the disease [2]. The five-year survival rate of AoV adenocarcinoma patients who underwent surgical resection was reported to be 30–60% [3]. However, the prognosis differs among previous studies, and the treatment outcomes are still unsatisfactory [4]. Therefore, it is clinically important to identify the prognostic factors to improve treatment outcomes.

Lymph node (LN) involvement is one of the well-demonstrated prognostic factors for AoV adenocarcinoma. Regional LN metastases are found in about 20–50% of patients with resected AoV adenocarcinoma patients and are related to worse survival [5–7]. In the current American Joint Committee on Cancer (AJCC) staging system, the N stage is defined according to the number of metastatic LNs: N0 (no regional LN metastasis), N1 (metastasis in 1–3 LNs), and N2 (metastasis in 4 or more LNs) [8]. Besides the number of metastatic LNs, various LN parameters are also suggested to predict prognosis. LN ratio (LNR) refers to the ratio of metastatic LN count to total harvested LNs and is associated with prognosis after curative resection in previous studies [9, 10]. Log odds of positive LNs (LODDS) is a recently introduced prognostic indicator [11]. It is considered an effective prognostic factor that comprehensively considers existing parameters such as the total number of metastatic and harvested LNs and LNR. According to previous studies, LODDS can be used as a prognostic factor in various gastrointestinal malignancies [11–14].

Evidence on the effect of adjuvant treatment in resected AoV adenocarcinoma patients is growing, and various LN parameters have been suggested to predict the prognosis. However, studies are still lacking in comparing the prognostic significance of each LN parameter in AoV adenocarcinoma patients who underwent surgical resection and adjuvant treatment.

In this study, we investigated the prognostic value of various LN parameters in AoV adenocarcinoma patients who underwent curative resection followed by adjuvant treatment. Through this study, we want to evaluate the prognostic value of LN parameters and identify a subgroup of patients who can benefit from adjuvant treatment.

## Methods

### Patients and enrollment criteria

We investigated patients who underwent curative resection followed by adjuvant treatment for AoV adenocarcinoma from January 2005 to February 2016. The exclusion criteria of this study were as follows: (1) patients who were diagnosed with malignancy other than AoV adenocarcinoma; (2) patients who were referred out to other hospitals and could not be sufficiently followed up; (3) patients diagnosed with another malignancy within five years after surgery; and (4) patients who underwent local resection without LN dissection, such as ampullectomy. We collected demographics, medical history, clinicopathologic findings, ongoing treatment, and clinical data during the follow-up period. Until December 2021, all eligible patients could have a sufficient follow-up period (at least five years) after curative resection.

In this study, surgeons and pathologists grouped the dissected LNs according to their anatomical location based on the definition provided by the Japanese Society of Hepato-Biliary-Pancreatic Surgery for ampullary region carcinoma [15]. LNs located on the anterior and posterior surfaces of the pancreas head, at the root of the mesenteric artery, around the bile duct within the hepatoduodenal ligament, along the common hepatic artery, and near the pyloric region were classified as regional LNs and were dissected. Non-regional LNs, including paraaortic nodes, were not routinely resected, but were removed if preoperative imaging could not distinguish between reactive and metastatic nodes, or if they appeared enlarged during intraoperative inspection, for example, during Kocher’s maneuver.

This study was approved by the institutional review board (IRB) of Seoul National University Hospital, and written informed consent was waived due to its retrospective nature (IRB approval number: H-1909-014-1061).

### Study outcomes and definitions

The primary endpoints are the overall survival (OS) and disease-free survival (DFS) of the patients. Locoregional recurrence-free survival (LRFS) and distant metastasis-free survival (DMFS) were investigated as secondary outcomes. All study outcomes were calculated from the date of curative operation of AoV adenocarcinoma to the date of events as follows: death from all causes (OS), recurrence at any sites (DFS), locoregional recurrences (LRFS), and distant recurrences (DMFS). We obtained the death data from the Korean Ministry of the Interior and Safety database. The recurrence of the disease in patients was monitored through follow-up evaluations, which included chest and abdomen CT scans every three months for the first year, every six months from the second to the fifth year, and annually thereafter as needed. Locoregional recurrence was defined as tumor recurrence in the tumor bed and the regional LN area [16]. Distant recurrence was defined as tumor recurrence other than locoregional recurrence. The pathologic TNM stage was determined by the 8th edition of the AJCC cancer staging manual [8].

### Determination of cut-off value for each LN parameter: LNN, LNR, LODDS

This study investigated the prognostic efficacy of various LN parameters: pN stage based on the AJCC 8th edition, presence of regional LN metastasis (pN stage based on the AJCC 7th edition), number of metastatic LN (LNN), LNR, and LODDS. LNR was defined as the ratio of the number of metastatic LN to total harvested LN, and LODDS was calculated using the following formula: log (LNN + 0.5 / total harvested LN – LNN + 0.5). The optimal cutoff values for continuous variables such as LNN, LNR, and LODDS were obtained using the maximal chi-square method to maximize the group difference, and the patients were dichotomized by these cutoff values and compared.

### Statistical analysis

The continuous variables are described with a median and interquartile range, and categorical variables are presented as numbers and percentages. Survival times and rates were estimated using the Kaplan–Meier method. A log-rank test was used to investigate the difference in survival between groups for each LN parameter.

To determine the prognostic factors associated with OS, DFS, LRFS, and DMFS, we performed a two-step Cox proportional hazards regression analysis. First, a univariable analysis was performed to identify the potential prognostic factors. Variables with statistical significance (*p*-value < 0.1) in the univariable analysis were subsequently analyzed using multivariable Cox regression models based on Akaike Information Criterion-based backward stepwise elimination [17]. Second, LNN, LNR, LODDS, pN stage, and LN involvement were separately analyzed using five different Cox regression models with the same confounders based on the result of the univariable analysis: LNN model, LNR model, LODDS model, N stage model, and LN involvement model.

All statistical analyses were performed using the R software environment (version 4.3.1; The R Foundation for Statistical Computing, Vienna, Austria), and a *p*-value lower than 0.05 was considered to be statistically significant.

## Results

### Study population and baseline clinicopathologic characteristics

Among a total of 459 patients who received surgical resection for periampullary tumors, 373 patients were excluded. The reasons for exclusion were as follows: 86 patients were diagnosed with other malignancies, 20 patients already had an unresectable malignancy at the time of diagnosis, 175 patients did not receive adjuvant treatment, 26 patients had insufficient follow-up periods, 16 patients were referred out to other hospitals, 20 patients were diagnosed with second-primary cancer within five years, and 30 patients received local resection without LN dissection (Supplementary Fig. 1).

The baseline clinicopathologic characteristics of all patients are described in Table 1. The median age of the patients was 59 years, and 46 (53.5%) of the patients were male. Most of the patients showed good performance statuses of Eastern Cooperative Oncology Group (ECOG-PS) 0 and 1 (96.5%). Fifty-one (59.3%) patients had advanced T stages (T3-4) by pathologic staging. LN metastasis was observed in 42 (48.9%) patients. Among them, 41 patients exhibited metastases confined to regional LNs. Specifically, 29 patients had metastases confined to LN surrounding the pancreatic head. Meanwhile, 12 patients demonstrated metastatic involvement of additional regional LNs, including those at root of the mesenteric artery, around the bile duct within the hepatoduodenal ligament, along the common hepatic artery, and near the pyloric region. The paraaortic LNs, considered non-regional nodes, were resected in a total of 15 patients, and metastasis was identified in one of them.

**Table 1:** Clinicopathologic characteristics of patients who underwent curative resection for ampullary adenocarcinoma

Characteristics	*N* = 86
Age	59.0 (55.0–64.0)
≥ 60	39 (45.3%)
< 60	47 (54.7%)
Gender	
Male	46 (53.5%)
Female	40 (46.5%)
Performance status (ECOG)	
0–1	83 (96.5%)
2	3 (3.5%)
Type of surgery	
PPPD	76 (88.4%)
Whipple	10 (11.6%)
Tumor differentiation	
Well differentiated	9 (10.5%)
Moderately differentiated	65 (75.6%)
Poorly differentiated	12 (13.9%)
Tumor size (cm)	2.1 (1.6–2.8)
Number of dissected LNs	15 (8–25)
pT stage	
1–2	35 (40.7%)
3–4	51 (59.3%)
pN stage	
0	44 (51.2%)
1	33 (38.4%)
2	9 (10.5%)
Microscopic lymphatic invasion	
Present	47 (54.7%)
Venous invasion	
Present	7 (8.1%)
Perineural invasion	
Present	24 (27.9%)
Preoperative CA 19-9^a^	
≤ 37	53 (61.6%)
> 37	33 (38.4%)
Postoperative CA 19-9^b^	
≤ 37	79 (91.9%)
> 37	7 (8.1%)
Adjuvant CCRT	75 (87.2%)
Additional chemotherapy after CCRT	
FL	1 (1.2%)
XP	1 (1.2%)

*ECOG* Eastern Cooperative Oncology Group, *LN* lymph node, *CA 19–9* carbohydrate antigen 19–9, *CCRT* concurrent chemoradiation therapy, *PPPD* pylorus preserving pancreaticoduodenectomy, *FL* fluorouracil and folinic acid, *XP* capecitabine and cisplatin

^a^Preoperative CA 19–9 refers to the value measured within one month before surgery, with the closest value to the surgery date

^b^Postoperative CA 19–9 refers to the CA 19–9 value measured at the first outpatient follow-up visit after recovery from surgery

Seventy-five (87.2%) patients received adjuvant concurrent chemoradiotherapy (CCRT). Among them, 70 patients received fluorouracil and five patients received capecitabine as a radiosensitizer, with all completing at least one month of treatment. Among the 11 patients who received adjuvant chemotherapy alone, eight received a combination of fluorouracil and leucovorin, and three received a combination of fluorouracil and cisplatin. Of these, seven patients completed six months of treatment, while four patients discontinued early. The adjuvant treatments were administered according to the clinicians’ decisions [18–26].

### Survival and patterns of cancer recurrence

The median follow-up period was 69.4 months, and the median OS was 114 months. The 1-, 2-, and 5-year survival rates were 96.5%, 88.4%, and 58.1%, respectively. During the follow-up period, disease recurrence occurred in 39 patients (45.3%), and most of the patients showed recurrence in distant organs. Local recurrence was identified in seven patients. Among them, six patients had a concurrent distant recurrence, and one patient was diagnosed with an isolated locoregional recurrence. Among the 38 patients with distant recurrence, the most common sites were the liver (24 patients, 63.2%), lungs (7 patients, 18.4%), and bones (5 patients, 13.2%). Other sites included distant LNs (8 patients, 21.1%), peritoneum (3 patients, 7.9%), brain (1 patient, 2.6%), hepatic hilum (1 patient, 2.6%), adrenal glands (1 patient, 2.6%), and remnant pancreas (1 patient, 2.6%). The 1-, 2-, and 5-year DFS rates were 82.6%, 66.2%, and 54.2%, respectively.

### Study outcomes according to each LN parameter

**Fig. 1:**
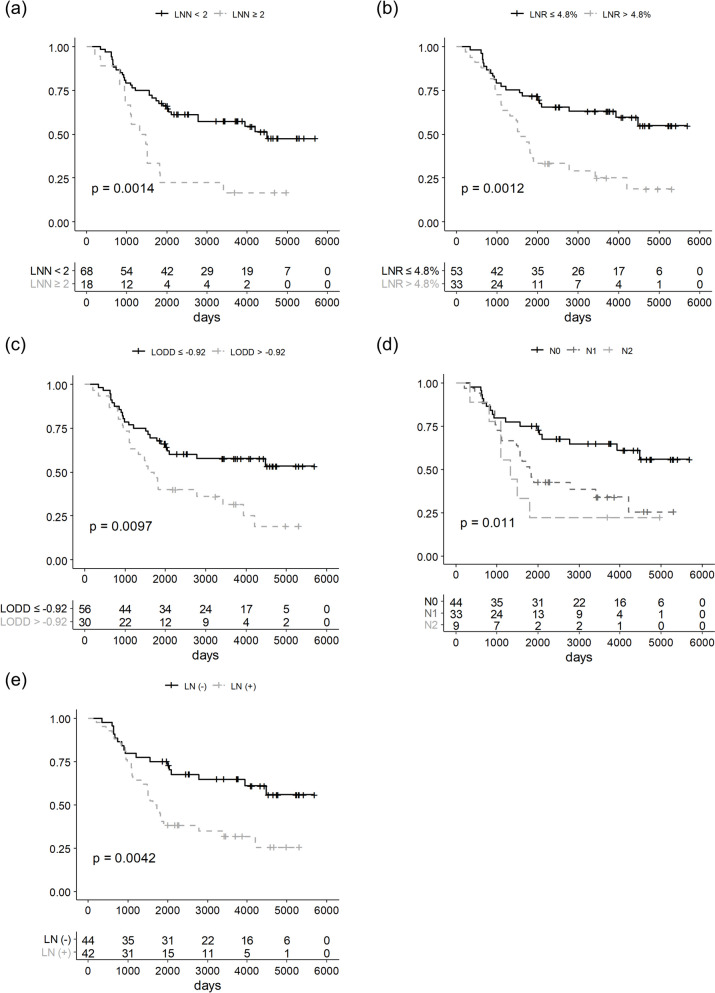
Kaplan–Meier plot of overall survival according to (**a**) LNN, **b** LNR, **c** LODDS, **d** pN stage, and **e** LN involvement

**Fig. 2:**
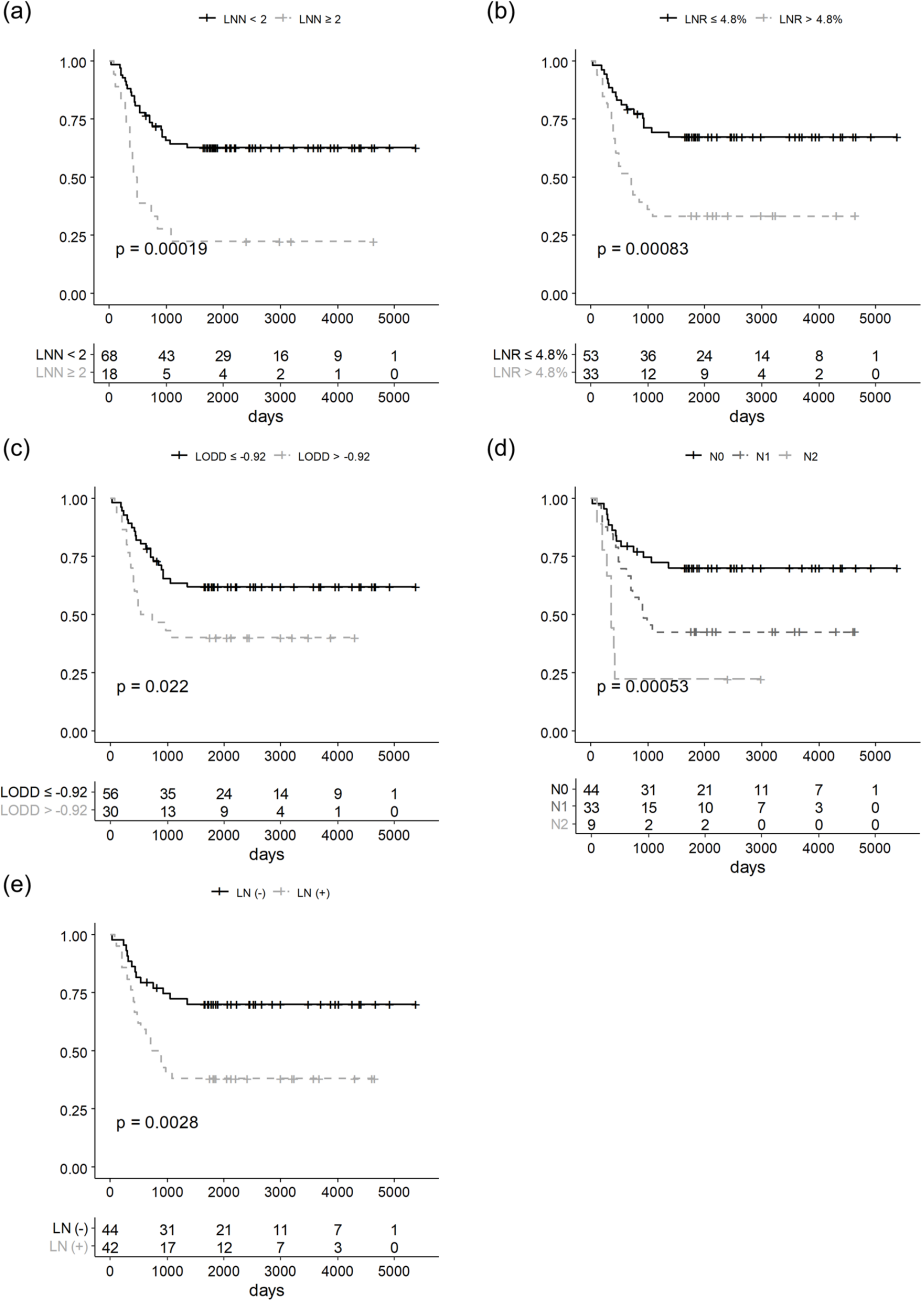
Kaplan–Meier plot of disease free-survival according to (**a**) LNN, **b** LNR, **c** LODDS, **d** pN stage, and **e** LN involvement

Each LN parameter, pN stage, presence of regional LN metastasis, LNN, LNR, and LODDS showed good prognostic efficacy for OS, DFS, and DMFS. The presence of regional LN metastasis and advanced pN stages was related to poor five-year OS, DFS, and DMFS. The optimal cutoff values of LNN, LNR, and LODDS were 1, 4.8%, and -0.92, respectively. When comparing survival after dichotomizing patients according to the cutoff value of each parameter, LNN (0–1 vs. ≥ 2), LNR (≤ 4.8% vs. > 4.8%), and LODDS (≤ -0.92 vs. > -0.92) showed good predictive efficacy in five-year OS, DFS, and DMFS. However, there is no LN parameter that is significantly associated with LRFS (Table 2). The results of Kaplan–Meier analyses for OS and DFS are demonstrated in Figs. 1 and 2, respectively. The difference in DMFS according to each LN parameter is also shown in Supplementary Fig. 2.

**Table 2:** Prognostic significance of various parameters related to lymph node status on each survival outcome

		N (%)	5-yr OS (%)	*P*-value^a^	5-yr DFS (%)	*P*-value^a^	5-yr LRFS (%)	*P*-value^a^	5-yr DMFS (%)	*P*-value^a^
LNN	0–1	68 (79.1)	67.6	0.001	62.8	< 0.001	89.7	0.18	76.8	< 0.001
	≥ 2	18 (20.9)	22.2		22.2		100		26.7	
LNR	≤ 4.8%	53 (61.6)	71.7	0.001	67.4	< 0.001	90.6	0.616	80.9	0.001^b^
	> 4.8%	33 (38.4)	36.4		33.3		93.6		42.4	
LODDS	≤ -0.92	56 (65.1)	67.9	0.01	61.8	0.022	91.1	0.744	74.2	0.042
	> -0.92	30 (34.9)	40		40		93		51.2	
pN stage	0	44 (51.2)	75	0.003	70	< 0.001	88.6	0.226	70	< 0.001
	1	33 (38.4)	45.5		42.4		93.8		44.3	
	2	9 (10.5)	22.2		22.2		100		22.2	
LN involvement	Absent	44 (51.2)	75	0.004	70	0.003	88.6	0.285	84	0.004
	Present	42 (48.8)	40.5		38.1		95.1		47.5	

*OS* overall survival, *DFS* disease-free survival, *LRFS* locoregional recurrence-free survival, *DMFS* distant metastasis-free survival, *LN* lymph node, *LNN* metastatic lymph node number, *LNR* metastatic lymph node ratio, *LODDS* log odds of positive lymph node

^a^*p*-Value by log-rank test

^b^Actual *p*-Value is 0.001460516

### Study outcomes analyses according to clinicopathologic characteristics

Table 3 summarizes the results of univariable analysis for prognostic factors associated with patients’ survival outcomes. Perineural invasion (PNI) and high post-operative carbohydrate antigen 19–9 (> 37 U/ml) were associated with poor OS, DFS, and DMFS. The patients with PNI showed worse five-year OS (29.2% vs. 69.4%; *p* = 0.002), DFS (27.5% vs. 64.3%; *p* < 0.001), and DMFS (30.0% vs. 64.3%; *p* = 0.001). The patients with higher post-operative carbohydrate antigen 19–9 showed significantly lower five-year OS (42.9% vs. 59.5%; *p* = 0.043), DFS (28.6% vs. 56.5%; *p* = 0.022), and DMFS (28.6% vs. 57.5%; *p* = 0.015). There were no clinical factors associated with LRFS in the univariable analysis.

**Table 3:** Univariable analysis identifying potential risk factors related to each survival outcome

		N (%)	5-yr OS (%)	*P*-value^a^	5-yr DFS(%)	*P*-value^a^	5-yr LRFS(%)	*P*-value^a^	5-yr DMFS(%)	*P*-value^a^
Age	≥ 60	39 (45.3)	61.7	0.058	57	0.517	95.6	0.167	57	0.653
	< 60	47 (54.7)	53.8		50.9		87.2		53	
Gender	Male	46 (53.5)	47.8	0.06	45.1	0.073	95.6	0.161	46.6	0.091
	Female	40 (46.5)	70		64.7		87.2		64.7	
Performance status (ECOG)	0–1	83 (96.5)	57.8	0.856	55	0.408	91.5	0.664	56	0.375
	2	3 (3.5)	66.7		33.3		100		33.3	
Type of surgery	PPPD	76 (88.4)	56.6	0.467	52.4	0.4	91.9	0.795	53.4	0.431
	Whipple	10 (11.6)	70		68.6		90		68.6	
Tumor differentiation	WD	9 (10.5)	88.9	0.061	87.5	0.050	100	0.352	87.5	0.056
	Non-WD	77 (89.5)	54.5		50.4		90.7		51.4	
pT stage	1–2	35 (40.7)	65.7	0.199	57.1	0.755	85.7	0.088	59.4	0.539
	3–4	51 (59.3)	52.9		52.1		96		52.1	
pN stage	0	44 (51.2)	75	0.003	70	< 0.001	88.6	0.226	70	< 0.001
	1	33 (38.4)	45.5		42.4		93.8		44.3	
	2	9 (10.5)	22.2		22.2		100		22.2	
Microscopic lymphatic invasion	Absent	39 (45.3)	69.2	0.070	63.7	0.096	89.7	0.560	63.7	0.122
	Present	47 (54.7)	48.9		46.3		93.5		47.8	
Venous invasion	Absent	79 (91.9)	60.8	0.153	55.4	0.210	91	0.434	56.4	0.182
	Present	7 (8.1)	28.6		42.9		100		42.9	
Perineural invasion	Absent	62 (72.1)	69.4	0.002	64.3	< 0.001	91.9	0.921	64.3	0.001
	Present	24 (27.9)	29.2		27.5		91.1		30	
Preoperative CA 19-9^b^	≤ 37	53 (61.6)	60.4	0.264	56.3	0.526	92.5	0.758	57.8	0.395
	> 37	33 (38.4)	54.5		51		90.5		51	
Postoperative CA 19-9c	≤ 37	79 (91.9)	59.5	0.043	56.5	0.022	92.4	0.364	57.5	0.015
	> 37	7 (8.1)	42.9		28.6		83.3		28.6	
Adjuvant CCRT	No	11 (12.8)	81.8	0.081	81.8	0.071	100	0.296	81.8	0.078
	Yes	75 (87.2)	54.7		50.1		90.5		51.1	

*OS* overall survival, *DFS* disease-free survival, *LRFS* locoregional recurrence-free survival, *DMFS* distant metastasis-free survival, *ECOG* Eastern Cooperative Oncology Group, *CA* 19–9 carbohydrate antigen 19–9, *CTx* chemotherapy, *CCRT* concurrent chemoradiation therapy, *WD* well differentiated carcinoma, *PPPD* pylorus-preserving pancreaticoduodenectomy

^a^*p*-Value by log-rank test

^b^Preoperative CA 19–9 refers to the value measured within one month before surgery, with the closest value to the surgery date

^c^Postoperative CA 19–9 refers to the CA 19–9 value measured at the first outpatient follow-up visit after recovery from surgery

There were no statistically significant differences in study outcomes based on the location of metastatic LNs. Among 41 patients with metastases confined to regional LNs, those with involvement limited to the LNs around the pancreatic head and those with metastases extending to other regional LNs exhibited comparable outcomes. The detailed results were as follows: OS (1619 days vs. 1654 days; *p* = 0.98), DFS (848 days vs. 622 days; *p* = 0.85), LRFS (1556 days vs. 1632 days; *p* = 0.36), and DMFS (902 days vs. 622 days; *p* = 0.97). Furthermore, the patient with paraaortic LN metastasis exhibited no recurrence for 2,990 days following surgery and no mortality for 4,973 days following surgery.

### Multivariable analysis of each model

To find out the prognostic efficacy of each LN parameter, multivariable analyses were performed on LNN, LNR, LODDS, pN stage, and LN involvement models. In the multivariable analyses for OS, only LNN ≥ 2 was significantly related to lower OS (Hazard ratio (HR) 2.10, 95% Confidence interval (CI) 1.11–3.97; *p* = 0.022). Other LN parameters, such as LNR (> 4.8%), LODDS (> -0.92), pN stage, and the presence of LN involvement, did not show a significant association with OS (Tables 4 and 5).

**Table 4:** Multivariable Cox regression analysis of LNN, LNR, and LODDS model for overall survival

			LNN model			LNR model			LODDS model	
		HR	95% CI	*P*	HR	95% CI	*P*	HR	95% CI	*P*
Age	< 60	1		0.007	1		0.020	1		0.006
	≥ 60	2.41	1.27–4.58		2.17	1.13–4.15		2.47	1.29–4.71	
Tumor differentiation	WD	1		0.122	1		0.114	1		0.122
	Non-WD	3.12	0.74–13.16		3.19	0.76–13.42		3.12	0.74–13.22	
Perineural invasion	Absent	1		< 0.001	1		< 0.001	1		< 0.001
	Present	3.47	1.79–6.72		3.12	1.60–6.06		3.40	1.75–6.59	
Adjuvant CCRT	No	1		0.120	1		0.136	1		0.113
	Yes	2.55	0.78–8.32		2.45	0.75–7.95		2.60	0.80–8.49	
LNN	0–1	1		0.022						
	≥ 2	2.10	1.11–3.97							
LNR	≤ 4.8%				1		0.088			
	> 4.8%				1.70	0.92–3.15				
LODDS	≤ -0.92							1		0.082
	> -0.92							1.69	0.93–3.06	

*LNN* metastatic lymph node number, *LNR* metastatic lymph node ratio, *LODDS* log odds of positive lymph node, *HR* hazard ratio, *CI* confidence interval, *CCRT* concurrent chemoradiation therapy, *WD* well differentiated carcinoma

**Table 5:** Multivariable Cox regression analysis of survival N stage and LN involvement model for overall survival

			N stage model		LN involvement model		
		HR	95% CI	*P*	HR	95% CI	*P*
Age	< 60	1		0.003	1		0.003
	≥ 60	2.65	1.40–5.02		2.65	1.40–5.02	
Tumor differentiation	WD	1		0.165	1		0.165
	Non-WD	2.85	0.65–12.44		2.85	0.65–12.44	
Microscopic lymphatic invasion	Absent	1		0.141	1		0.141
	Present	1.63	0.85–3.10		1.63	0.85–3.10	
Perineural invasion	Absent	1		< 0.001	1		< 0.001
	Present	3.37	1.77–6.43		3.37	1.77–6.43	
Adjuvant CCRT	No	1		0.112	1		0.112
	Yes	2.61	0.80–8.54		2.63	0.80–8.54	
pN stage	0						
	1						
	2						
LN involvement	Absent						
	Present						

*LN* lymph node, *HR* hazard ratio, *CI* confidence interval, *CCRT* concurrent chemoradiation therapy, *WD* well differentiated carcinoma

In the multivariable analyses for DFS, LNN ≥ 2 (HR 2.51, 95% CI 1.28–4.93; *p* = 0.007) and LNR > 4.8% (HR 2.35, 95% CI 1.23–4.50; *p* = 0.010) were significantly related to shorter DFS. Patients with the pN2 stage showed worse OS than those with pN0 (HR 4.31, 95% CI 1.66–11.20; *p* = 0.003), but there was no statistically significant difference in DFS between the pN1 and pN0 stages (HR 1.55, 95% CI 0.74–3.26; *p* = 0.243). LODDS and the presence of LN metastasis were not significantly associated with DFS. The result of the multivariable analysis for DFS is summarized in Tables 6 and 7.

**Table 6:** Multivariable Cox regression analysis of LNN, LNR, and LODDS model for disease-free survival

			LNN model			LNR model			LODDS model	
		HR	95% CI	*P*	HR	95% CI	*P*	HR	95% CI	*P*
Tumor differentiation	WD	1		0.125	1		0.113	1		0.072
	Non-WD	4.82	0.65–36.04		5.06	0.68–37.59		6.26	0.85–46.13	
Perineural invasion	Absent	1		0.002	1		0.001	1		< 0.001
	Present	2.79	1.46–5.32		2.88	1.51–5.50		3.02	1.58–5.77	
Postoperative	≤ 37	1		0.050^b^				1		0.057
CA 19–9^a^	> 37	2.67	1.00–7.12					2.62	0.97–7.07	
Adjuvant CCRT	No	1		0.090	1		0.155	1		0.067
	Yes	3.57	0.82–15.52		2.83	0.68–11.81		4.01	0.91–17.72	
LNN	0–1	1		0.007						
	≥ 2	2.51	1.28–4.93							
LNR	≤ 4.8%				1		0.010			
	> 4.8%				2.35	1.23–4.50				
LODDS	≤ -0.92									
	> -0.92									

*LNN* metastatic lymph node number, *LNR* metastatic lymph node ratio, *LODDS* log odds of positive lymph node, *HR* hazard ratio, *CI* confidence interval, *CCRT* concurrent chemoradiation therapy, *CA 19–9* carbohydrate antigen 19–9, *WD* well differentiated carcinoma

^a^Postoperative CA 19–9 refers to the CA 19–9 value measured at the first outpatient follow-up visit after recovery from surgery

^b^*p*-Value is greater than 0.050

**Table 7:** Multivariable Cox regression analysis of N stage and LN involvement model for disease-free survival

			N stage model		LN involvement model		
		HR	95% CI	*P*	HR	95% CI	*P*
Tumor differentiation	WD	1		0.110	1		0.093
	Non-WD	5.14	0.69–38.24		5.56	0.75–41.12	
Perineural invasion	Absent	1		0.001	1		0.002
	Present	2.96	1.53–5.72		2.78	1.45–5.32	
Postoperative	≤ 37	1		0.073	1		0.121
CA 19–9^a^	> 37	2.51	0.92–6.88		2.17	0.81–5.79	
Adjuvant	No	1		0.128	1		0.137
CCRT	Yes	3.18	0.72–14.15		3.05	0.70–13.27	
pN stage	0	1					
	1	1.55	0.74–3.26	0.243			
	2	4.31	1.66–11.20	0.003			
LN involvement	Absent				1		0.068
	Present				1.91	0.95–3.82	

*LN* lymph node, *HR* hazard ratio, *CI* confidence interval, *CCRT* concurrent chemoradiation therapy, *CA 19–9* carbohydrate antigen 19–9, *WD* well differentiated carcinoma

^a^Postoperative CA 19–9 refers to the CA 19–9 value measured at the first outpatient follow-up visit after recovery from surgery

The result of the multivariable analyses for DMFS was similar to that for DFS (Supplementary Table 1). LNN ≥ 2 (HR 2.74, 95% CI 1.39–5.41; *p* = 0.004), LNR > 4.8% (HR 2.26, 95% CI 1.17–4.35; *p* = 0.015), and pN2 stage (HR 4.59, 95% CI 1.76–11.97; *p* = 0.002) showed worse DMFS. LODDS and the presence of LN metastasis did not show a significant association with DMFS in each model. There were no factors associated with LRFS in the univariable analysis, so a multivariable analysis for LRFS was not performed.

## Discussion

This study investigated the prognostic efficacy of each LN parameter, and the involvement of two or more regional LN (LNN ≥ 2) is a significant prognostic factor for AoV cancer patients who underwent a curative operation and adjuvant treatment. LNN ≥ 2 showed consistent prognostic performance in both univariable and multivariable analyses for OS, DFS, and DMFS. LNR > 4.8% was also a significant factor for predicting DFS, and PNI was significantly related to OS and DFS.

Several studies reported that an increased number of metastatic regional LNs was associated with poor survival outcomes [6, 27, 28]. Therefore, in the AJCC staging system, the N stage of resected AoV adenocarcinoma was revised from absent (N0) or present (N1) regional LN metastasis (7th edition) to N0 (no regional LN metastasis), N1 (1–3 LNs), and N2 (4 or more LNs) (8th edition). This study also showed the prognostic performance of pN stages in the AJCC 7th and 8th editions in the univariable analysis. However, the multivariable analyses for OS, DFS, and DMFS showed that LNN ≥ 2 has better predictive power than pN stages. Considering that this study analyzed patients who received adjuvant treatment and that the majority (95.3%) of them completed the planned adjuvant treatment, it can be assumed that completion of the planned adjuvant treatment may help prevent recurrence in patients with a small number of regional LN metastases [29].

Despite potential advantages that might prevent a recurrence, the benefit of adjuvant treatment in AoV adenocarcinoma remains controversial. In the previous phase 3 randomized trial (ESPAC-3 trial), adjuvant chemotherapy showed a statistically significant survival benefit in multivariable analysis [24]. However, in the same study, the survival benefit of adjuvant chemotherapy was not confirmed in the primary analysis, and recent retrospective studies of resected AoV adenocarcinoma patients showed that adjuvant chemotherapy did not improve the survival of patients [5, 30]. The benefit of adjuvant radiotherapy is also still controversial [31]. In this study population, in which most patients underwent adjuvant CCRT, the median OS was 9.5 years, and the five-year OS rate was 58.1%. In particular, compared with the previous studies, which reported local recurrence rates of 14 to 31%, the local recurrence rate was very low at 8.6% [5, 14, 32, 33], and there was no parameter related to LRFS. The reason for the low local recurrence rate in this population could be that most of the margin-positive (R1) resection patients were excluded during enrollment. Also, the potential role of adjuvant CCRT in locoregional control cannot be negligible [34].

In univariable analysis, LNR and LODDS showed significant relationships with five-year OS, DFS, and DMFS. However, in the multivariable analysis including clinicopathologic factors, the clinical significance of LNR and LODDS was lost. The pN stages in the 7th and 8th AJCC editions also showed the same pattern. Among the patients with high LNR (> 4.8%, *n* = 33) and LODDS > -0.92 (*n* = 30), who were considered to have a poor prognosis, 45.5% (15 of 33) and 30.0% (9 of 30) patients had one metastatic LN. Patients with one regional LN metastasis showed better OS (78 months vs. 47 months, *p* = 0.071), DFS (33 months vs. 15 months, *p* = 0.032), and DMFS (not reached vs. 15 months, *p* = 0.018) than LNN ≥ 2 patients. Considering that the differences in OS, DFS, and DMFS in prognosis between LNN = 1 and LNN = 0 patients were not significant, a substantial proportion of LNN = 1 patients in the bad prognosis group may have diminished the clinical significance of LNR and LODDS.

In addition, the clinical significance of LNR and LODDS may have decreased due to the influence of PNI, which is a significant prognostic factor in multivariable analyses of all models. PNI can be diagnosed when the tumor is located close to the nerve and surrounds at least one-third of the circumference, or when tumor cells invade any of the three layers of the nerve sheath [35]. PNI is recognized as a metastatic route and is related to the poor prognosis of various gastrointestinal cancers [36–39]. In this study population, 24 (27.9%) patients had PNI and showed worse five-year OS (29.2 vs. 69.4 months; *p* = 0.002), DFS (27.5 vs. 64.3 months; *p* < 0.001), and DMFS (30 months vs. 64.3 months; *p* = 0.001) than negative PNI patients. Thus, PNI, as a strong predictor, overwhelmed LN parameters except for LNN in the multivariable Cox regression model. In particular, distant metastasis was developed in most of the PNI ( +) patients (16 of 24 patients, 66.7%) regardless of the negative margin status and adjuvant CCRT, which could contribute to local control. This suggests that PNI can be a systemic spread route in AoV adenocarcinoma, like in other malignancies. Therefore, patients with PNI may require intensive post-operative treatment and follow-up regardless of regional LN status.

This study has several limitations. First, this is a single-center retrospective study that has inevitable selection biases. Only patients with obvious medical records about the operation, adjuvant treatment, and follow-up were included. Patients with arbitrary follow-up losses, patients who transferred out to other hospitals, and patients with the development of second-primary malignancy were excluded. Therefore, a relatively small number of patients were analyzed, which has limitations in deriving a solid conclusion. Second, cancer-associated deaths could not be analyzed because some clinical information, including the cause of death, was not accessible in the government data. However, the government database provides complete information about the time of death and is sufficient for analyzing the OS.

Despite its limitations, this study has strengths, as it was a long-term study conducted with a homogeneous patient population. This study presented reliable long-term follow-up data of AoV adenocarcinoma patients who underwent curative resection followed by adjuvant treatment. In this population, it showed that adjuvant treatment might improve the prognosis of patients with oligometastasis of regional LN. Also, it presented implications for the utilization of various LN parameters.

## Conclusion

LN metastasis is a significant prognostic factor in AoV adenocarcinoma patients who underwent curative resection followed by adjuvant chemotherapy. Considering the result of multivariable analyses, LNN (0–1 vs. ≥ 2) could show better prognostic power in patients who received adjuvant treatment. Further large-scale studies are needed to validate the clinical usefulness of various LN parameters in AoV adenocarcinoma patients with postoperative treatment.

## Supplementary Information

Supplementary Material 1.

## Data Availability

No datasets were generated or analysed during the current study.

## Abbreviations

LN: Lymph node
AoV: Ampulla of Vater
LNN: Lymph node number
LNR: Lymph node ratio
LODDS: Log odds of positive lymph nodes
OS: Overall survival
DFS: Disease-free survival
DMFS: Distant metastasis-free survival
HR: Hazard ratio
CI: Confidence interval
AJCC: American Joint Committee on Cancer
LRFS: Locoregional recurrence-free survival
CCRT: Concurrent chemoradiotherapy
PNI: Perineural invasion
