# Supplementary material for: Prognostic efficacy of lymph node parameters in resected ampullary adenocarcinoma based on long-term follow-up data after adjuvant treatment
Source: World J Surg Oncol. 2024 Nov 21;22:308. doi: 10.1186/s12957-024-03587-z (PMC11580443; doi:10.1186/s12957-024-03587-z)
Supplement: Supplementary file 1 — Supplementary Material 1. [file 12957_2024_3587_MOESM1_ESM.docx]

Supplementary Table 1. Multivariable Cox regression analysis of each model for distant metastasis-free survival: (a) LNN, LNR, and LODDS model, (b) N stage and LN involvement model

(a)

|  |  |  | LNN model |  |  |  | LNR model |  |  |  | LODDS model |  |  |
| --- | --- | --- | --- | --- | --- | --- | --- | --- | --- | --- | --- | --- | --- |
|  |  | HR | 95% CI | *P* |  | HR | 95% CI | *P* |  | HR | 95% CI | *P* |  |
| Tumor | WD | 1 |  | 0.144 |  | 1 |  | 0.119 |  | 1 |  | 0.080 |  |
| differentiation | Non-WD | 4.48 | 0.60-33.55 |  |  | 4.93 | 0.66-36.66 |  |  | 5.95 | 0.81-43.86 |  |  |
| Perineural | Absent | 1 |  | 0.004 |  | 1 |  | 0.003 |  | 1 |  | 0.002 |  |
| invasion | Present | 2.64 | 1.37-5.09 |  |  | 2.70 | 1.40-5.22 |  |  | 2.86 | 1.48-5.52 |  |  |
| Postoperative | ≤37 | 1 |  | 0.033 |  |  |  |  |  | 1 |  | 0.035 |  |
| CA 19-9^*^ | >37 | 2.92 | 1.09-7.81 |  |  |  |  |  |  | 2.92 | 1.08-7.90 |  |  |
| Adjuvant | No | 1 |  | 0.091 |  | 1 |  | 0.161 |  | 1 |  | 0.065 |  |
| CCRT | Yes | 3.56 | 0.82-15.49 |  |  | 2.79 | 0.67-11.65 |  |  | 4.07 | 0.92-18.09 |  |  |
| LNN | 0-1 | 1 |  | 0.004 |  |  |  |  |  |  |  |  |  |
|  | ≥2 | 2.74 | 1.39-5.41 |  |  |  |  |  |  |  |  |  |  |
| LNR | ≤4.8% |  |  |  |  | 1 |  | 0.015 |  |  |  |  |  |
|  | >4.8% |  |  |  |  | 2.26 | 1.17-4.35 |  |  |  |  |  |  |
| LODDS | ≤-0.92 |  |  |  |  |  |  |  |  |  |  |  |  |
|  | >-0.92 |  |  |  |  |  |  |  |  |  |  |  |  |

(b)

|  |  |  | N stage model |  |  | LN involvement model | | |  |
| --- | --- | --- | --- | --- | --- | --- | --- | --- | --- |
|  |  | HR | 95% CI | *P* |  | HR | 95% CI | *P* |  |
| Tumor | WD | 1 |  | 0.121 |  | 1 |  | 0.102 |  |
| differentiation | Non-WD | 4.89 | 0.66-36.40 |  |  | 5.31 | 0.72-39.31 |  |  |
| Perineural | Absent | 1 |  | 0.002 |  | 1 |  | 0.004 |  |
| invasion | Present | 2.85 | 1.45-5.57 |  |  | 2.63 | 1.36-5.09 |  |  |
| Postoperative | ≤37 | 1 |  | 0.043 |  | 1 |  | 0.077 |  |
| CA 19-9^*^ | >37 | 2.85 | 1.03-7.85 |  |  | 2.43 | 0.91-6.50 |  |  |
| Adjuvant | No | 1 |  | 0.121 |  | 1 |  | 0.132 |  |
| CCRT | Yes | 3.28 | 0.73-14.76 |  |  | 3.11 | 0.71-13.60 |  |  |
| pN stage | 0 | 1 |  |  |  |  |  |  |  |
|  | 1 | 1.46 | 0.69-3.10 | 0.323 |  |  |  |  |  |
|  | 2 | 4.59 | 1.76-11.97 | 0.002 |  |  |  |  |  |
| LN involvement | Absent |  |  |  |  | 1 |  | 0.087 |  |
|  | Present |  |  |  |  | 1.84 | 0.91-3.71 |  |  |

*LNN*, metastatic lymph node number; *LNR*, metastatic lymph node ratio; *LODDS*, log odds of positive lymph node; *LN*, lymph node; *HR*, hazard ratio; *CI*, confidence interval; *CCRT*, concurrent chemoradiation therapy; *CA 19-9*, carbohydrate antigen 19-9; *WD*, well differentiated carcinoma.

* Postoperative CA 19-9 refers to the CA 19-9 value measured at the first outpatient follow-up visit after recovery from surgery.

Supplementary Figure 1. Flowchart of patient enrollment


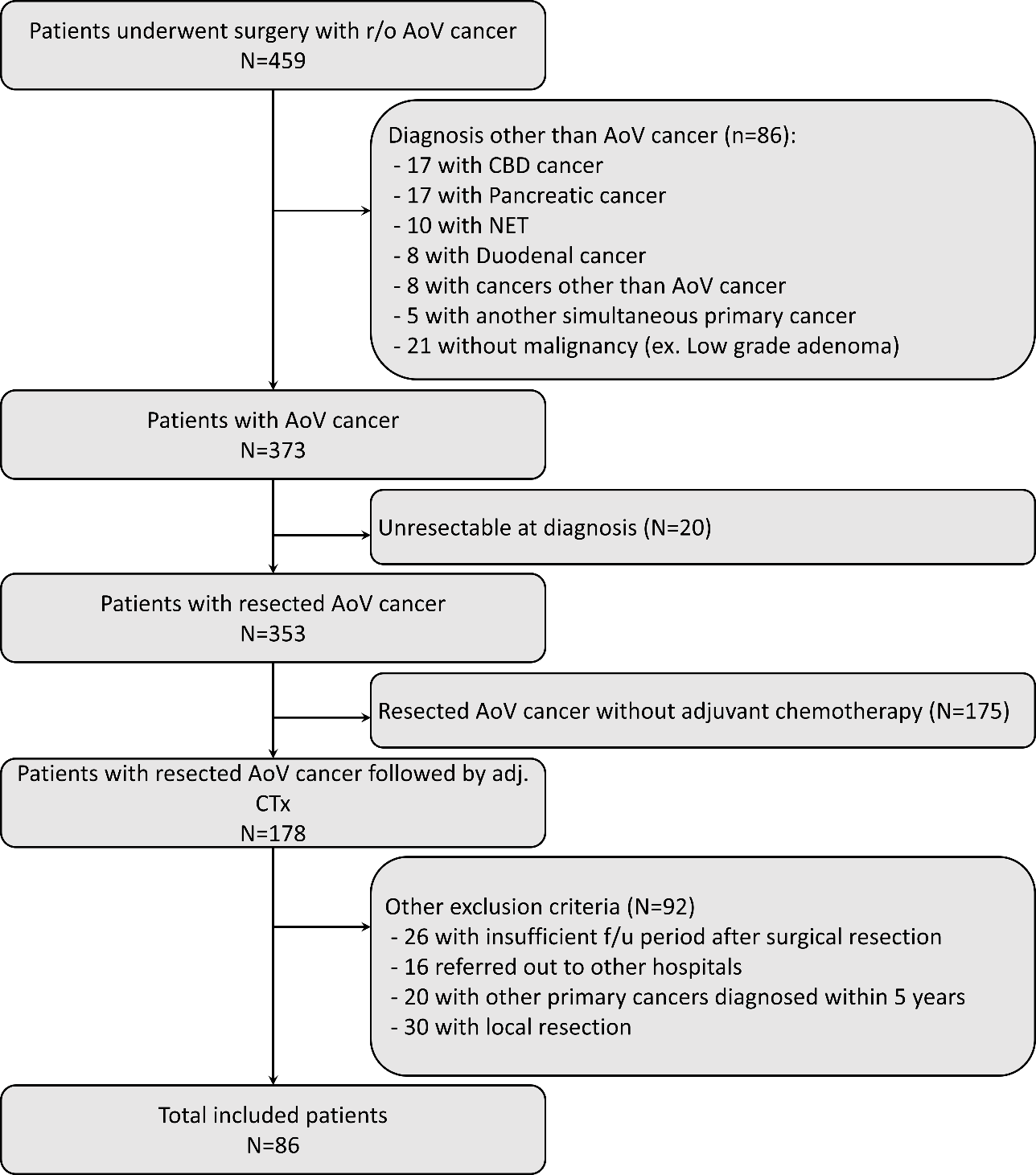


Supplementary Figure 2. Kaplan-Meier plot of distant metastasis-free survival according to (a) LNN, (b) LNR, (c) LODDS, (d) pN stage, and (e) LN involvement

(a) (b)

 (c) (d)

 (e)

*LNN*, metastatic lymph node number; *LNR*, metastatic lymph node ratio; *LODDS*, log odds of positive lymph node; *LN*, lymph node.
